# Dietary and Plasma Magnesium and Risk of Coronary Heart Disease Among Women

**DOI:** 10.1161/JAHA.113.000114

**Published:** 2013-04-24

**Authors:** Stephanie E. Chiuve, Qi Sun, Gary C. Curhan, Eric N. Taylor, Donna Spiegelman, Walter C. Willett, JoAnn E. Manson, Kathryn M. Rexrode, Christine M. Albert

**Affiliations:** 1 Center for Arrhythmia Prevention, Department of Medicine Brigham and Women's Hospital and Harvard Medical School, Boston, MA (S.E.C., C.M.A.); 2 Division of Preventive Medicine, Department of Medicine Brigham and Women's Hospital and Harvard Medical School, Boston, MA (S.E.C., J.A.E.M., K.M.R., C.M.A.); 3 Cardiovascular Division, Department of Medicine Brigham and Women's Hospital and Harvard Medical School, Boston, MA (C.M.A.); 4 Renal Division, Department of Medicine Brigham and Women's Hospital and Harvard Medical School, Boston, MA (G.C.C.); 5 The Channing Division for Network Medicine, Department of Medicine Brigham and Women's Hospital and Harvard Medical School, Boston, MA (Q.S., G.C.C., E.N.T., W.C.W.); 6 Department of Nutrition, Harvard School of Public Health, Boston, MA (S.E.C., Q.S., W.C.W.); 7 Department of Epidemiology, Harvard School of Public Health, Boston, MA (G.C.C., D.S., W.C.W., J.A.E.M.)

**Keywords:** epidemiology, myocardial infarction, nutrition, prevention

## Abstract

**Background:**

Magnesium is associated with lower risk of sudden cardiac death, possibly through antiarrhythmic mechanisms. Magnesium influences endothelial function, inflammation, blood pressure, and diabetes, but a direct relation with coronary heart disease (CHD) risk has not been established.

**Methods and Results:**

We prospectively examined the association between dietary and plasma magnesium and risk of CHD among women in the Nurses' Health Study. The association for magnesium intake was examined among 86 323 women free of disease in 1980. Information on magnesium intake and lifestyle factors was ascertained every 2 to 4 years through questionnaires. Through 2008, 3614 cases of CHD (2511 nonfatal/1103 fatal) were documented. For plasma magnesium, we conducted a nested case–control analysis, with 458 cases of incident CHD (400 nonfatal/58 fatal) matched to controls (1:1) on age, smoking, fasting status, and date of blood sampling. Higher magnesium intake was not associated with lower risk of total CHD (*P*‐linear trend=0.12) or nonfatal CHD (*P*‐linear trend=0.88) in multivariable models. However, magnesium intake was inversely associated with risk of fatal CHD. The RR comparing quintile 5 to quintile 1 of magnesium intake was 0.61 (95% CI, 0.45 to 0.84; *P*‐linear trend=0.003). The association between magnesium intake and risk of fatal CHD appeared to be mediated partially by hypertension. Plasma magnesium levels above 2.0 mg/dL were associated with lower risk of CHD, although not independent of other cardiovascular biomarkers (RR, 0.67; 95% CI, 0.44 to 1.04).

**Conclusions:**

Dietary and plasma magnesium were not associated with total CHD incidence in this population of women. Dietary magnesium intake was inversely associated with fatal CHD, which may be mediated in part by hypertension.

## Introduction

Magnesium is a principal intracellular cation in human cells and is an activator of >300 enzymes, most notably, sodium potassium ATPase.^1^ Thus, magnesium plays a vital role in numerous diverse cellular processes, including cardiovascular pathways. Previously, we reported a strong inverse association between dietary and plasma magnesium and risk of sudden cardiac death (SCD) in the Nurses' Health Study (NHS).^2^ We hypothesized that this association might be explained, in part, by the antiarrhythmic properties of magnesium.^3^ However, magnesium also has beneficial effects on traditional coronary heart disease (CHD) risk factors such as blood pressure,^4^ diabetes,^5^ and lipids,^6^ which might also explain part of the association with SCD. Further, magnesium may also lower risk of CHD directly through these atherosclerotic risk factors; however, a direct relationship between serum and/or dietary magnesium and CHD risk has not been clearly established. Serum and dietary magnesium have been inversely associated with CHD risk in some^7–9^ but not all^10–11^ epidemiological studies, and few studies have examined the association for serum and dietary magnesium within the same population.

To address these uncertainties, we examined the association between dietary and plasma magnesium and risk of CHD among middle‐aged and older women in the NHS. In addition, to evaluate whether the association between magnesium and CHD could be explained fully through traditional cardiovascular pathways, we quantified the degree to which its association with CHD was mediated by hypertension, diabetes, and hypercholesterolemia.

## Methods

### Study Population

The NHS began in 1976 and consists of 121 700 female nurses 30 to 55 years old at baseline.^12^ Every 2 years, information on lifestyle habits, cardiovascular disease (CVD) risk factors, and newly diagnosed diseases is ascertained through self‐administered questionnaires. Between 1989 and 1990, 32 826 women in this cohort provided a blood sample. The study was approved by the institutional review board at Brigham and Women's Hospital. Return of the baseline questionnaire implied informed consent.

### End‐Point Ascertainment and Definitions

Incident CHD was defined as nonfatal myocardial infarction (MI) or fatal CHD. MIs were defined by WHO criteria.^13^ Fatal CHD was defined as a fatal MI, according to autopsy or hospital records, or if CHD was listed as the cause of death and other evidence of CHD was present. We also included fatal events presumed to be CHD based on a death certificate alone. All CHD cases were confirmed by study physicians blinded to participants' risk factor status.

### Assessment of Magnesium Intake

Information on usual diet was ascertained via food frequency questionnaires (FFQs) in 1980, 1984, and every 4 years from 1986 to 2006. For each food item, each participant was asked how often, on average, she had consumed a specified portion size over the past year. We calculated the average intake of all nutrients by multiplying the frequency of consumption of each food by its nutrient content in the Harvard University Food Composition Database and summing across all foods. Total nutrient intake also included supplemental sources. For magnesium, supplements accounted for an average of 14 mg/day. The Pearson correlation coefficient between magnesium intake estimated from the FFQ and the average of two 1‐week dietary records was 0.67.^14^ All nutrients were adjusted for total energy intake using the residual method.^15^

### Measurement of Biochemical Variables

Procedures for blood collection and storage have been described previously.^16^ Plasma magnesium was measured by a colorimetric assay (Roche Diagnostics, Indianapolis, IN). The coefficient of variation (CV) was 4%. We measured total cholesterol, high‐density lipoprotein cholesterol (HDL‐C) and low‐density lipoprotein cholesterol (LDL‐C), high‐sensitivity C‐reactive protein (hsCRP), adiponectin, and hemoglobin A1c (HbA1c), as described previously.^17^ The CVs were <15% for adiponectin and <5% for other biomarkers. We used plasma creatinine (CV=12.8%), measured by a modified Jaffe method, to estimate the glomerular filtration rate (eGFR), based on the Modification of Diet in Renal Disease Study Group equation.

### Statistical Analysis

#### Dietary magnesium

The association between magnesium intake and CHD was analyzed in a prospective cohort design among the 86 323 women free of CHD and cancer and who provided valid dietary data (ie, <10 food items blank and total energy intake between 600 and 3500 kcal/day).

We calculated the cumulative average of total energy and magnesium and other nutrients to best represent long‐term diet and to reduce measurement error.^18^ For CHD incidence during 1984–1986, we used the average of magnesium intake in 1980 and 1984, for CHD incidence during 1986–1990, we used the average magnesium intake in 1980, 1984, and 1986, and so forth.

Dietary changes can occur after the development of intermediate end points in the causal pathway between magnesium intake and CHD, such as hypercholesterolemia, hypertension, and diabetes, which may then confound the association. Therefore, we assessed whether a diagnosis of these intermediate events was associated with a subsequent change in magnesium intake.^19^ If we detected a significant change after the diagnosis of the intermediate event, we stopped updating magnesium intake to avoid time‐dependent confounding by the intermediate event. If magnesium intake was not significantly different, we continued to update magnesium intake to avoid misclassification of the participants' long‐term magnesium intake. This approach represents our best efforts to account for confounding by intermediate events and to minimize misclassification of an individual's long‐term diet.

Women contributed person‐time from date of return of the 1980 questionnaire until the date of CHD diagnosis, date of death, or June 1, 2008, whichever came first. We calculated the hazard ratio (95% CI) of CHD by quintiles of magnesium intake using multivariable Cox proportional hazard models as an estimate of the relative risk (RR), adjusting for potential confounders. Magnesium intake and other covariates, except for parental history of MI, were updated during follow‐up and included as time‐varying covariates in the models. In separate models, we adjusted for history of diabetes, hypertension, and hypercholesterolemia at baseline and development of these intermediate diseases during follow‐up (as time‐varying covariates). Hypertension was defined as self‐reported physician diagnosis of disease and/or reported use of anti‐hypertensive medication. To test for a linear trend, we assigned each quintile the median value and modeled this variable as a continuous variable. We examined potential deviation from linearity with a likelihood ratio test, comparing a model with the linear term with a model including the linear term plus restricted cubic spline transformations.^20^ The assumption of proportional hazards was met.

To quantify the potential mediating effect of intermediate diseases (hypertension, diabetes, and high cholesterol), we calculated the mediation proportion, which is the proportion of the protective effect of magnesium intake on CHD risk that can be explained by associations with hypertension, diabetes, and hypercholesterolemia. For these models, information on these covariates was updated during follow‐up and included as time‐varying covariates in the models. We then estimated the change in beta‐coefficient for a 100‐mg increment of magnesium intake, comparing models with and without potential intermediates.

#### Plasma magnesium

We used a prospective nested case–control design to estimate the association between plasma magnesium and risk of CHD. Among the women who provided a blood sample and were free of CVD, 458 cases of CHD (400 nonfatal/58 fatal) occurred after return of the blood sample and before June 1, 2006. We randomly selected 1 control for each case among women free of CVD at the time of case diagnosis, matched on age (±1 year), smoking status (never/past/current), fasting status (yes/no), and month of blood draw. Magnesium and other assays were performed on the case–control pairs in the same analytical run, with laboratory personnel blinded to case status.

Age‐adjusted Spearman correlations quantified the association between plasma and dietary magnesium. Plasma magnesium was categorized into quartiles based on the distribution among the control participants. We used conditional logistic regression to estimate the relative risk (95% CI) of plasma magnesium in relation to CHD incidence. With a risk‐set sampled case–control design, the odds ratio estimated from the logistic regression model validly estimates the incidence ratio^21^ and thus the relative risk. Models were further adjusted for potential confounders ascertained in 1990, the year of blood collection. Tests for linear trend, deviation from linearity, and mediation proportion estimates per 1 standard deviation increment of plasma magnesium (0.2 mg/dL) were performed as described above. All statistical analysis was performed using SAS software (version 9). A *P* value <0.05 was considered statistically significant.

## Results

### Dietary Magnesium Analysis

Women with higher magnesium intake tended to be older; more likely to smoke, take multivitamins, and be more physically active; have higher intake of potassium, vitamin D, polyunsaturated:saturated fat, and cereal fiber; and have lower intake of trans fats (Table 1). Over a median follow‐up of 28 years, we documented 3614 cases of CHD (2511 nonfatal/1103 fatal events). Higher magnesium intake was associated with lower risk of total CHD in models adjusting for potential confounders (Table 2**,** model 1), but this association was attenuated and no longer significant after further adjustment for hypertension, diabetes, and hypercholesterolemia at baseline (Table 2, model 2). This attenuation was driven primarily by hypertension. Finally, results were consistent in age‐stratified analysis. The RR of total CHD, comparing quintile 5 with quintile 1 of dietary magnesium, was 0.86 (95% CI, 0.61 to 1.22) among women <60 years and 0.90 (95% CI, 0.74 to 1.11) for women ≥60 years.

**Table 1.** Baseline Characteristics* Among Women in the Nurses' Health Study by Quintile (Q) of Magnesium Intake

	Magnesium Intake				
	Q1	Q2	Q3	Q4	Q5
Range of magnesium, mg/day	<246	246 to 276	277 to 305	306 to 342	>342
n	21 867	15 988	14 955	15 279	18 234
Age, y	45 (7)*	46 (7)	47 (7)	47 (7)	48 (7)
Current smoker, %	27	28	29	30	31
BMI, kg/m^2^	24.7 (4.9)	24.4 (4.6)	24.2 (4.3)	24.2 (4.2)	24.2 (4.2)
Physical activity, h/week	3.5 (2.8)	3.7 (2.9)	3.9 (2.9)	4.1 (2.9)	4.4 (2.9)
Current use of hormone therapy, %	8	8	8	8	8
Multivitamin use, %	29	32	35	36	39
Aspirin use, %	14	14	14	15	15
Nutrients					
Potassium, mg/day	2123 (325)	2535 (294)	2769 (327)	3007 (366)	3466 (523)
Vitamin D, IU/day	267 (248)	304 (262)	332 (280)	353 (292)	399 (318)
Trans fat, g/day	4.5 (1.4)	4.2 (1.2)	4.0 (1.2)	3.9 (1.3)	3.4 (1.3)
Polyunsaturated:saturated fat	0.33 (0.12)	0.34 (0.12)	0.34 (0.12)	0.35 (0.13)	0.38 (0.16)
Cereal fiber, g/day	2.1 (1.3)	2.4 (1.4)	2.5 (1.5)	2.6 (1.6)	2.8 (1.7)
Alcohol, g/day	5.8 (10.8)	6.2 (10.2)	6.6 (10.2)	6.8 (10.7)	6.9 (10.8)
History of comorbidities, %					
Hypertension	17	16	15	15	15
Diabetes	2	2	2	2	2
High cholesterol	5	5	5	5	6

BMI indicates body mass index; SD, standard deviation.

* All nutrients except alcohol were energy adjusted.

* Mean (SD).

**Table 2.** Relative Risk (95% CI) of Total, Nonfatal, and Fatal CHD by Quintile (Q) of Magnesium Intake

	Magnesium Intake					*P*–Linear Trend*
	Q1	Q2	Q3	Q4	Q5	
Range of magnesium, mg/day	<246	246 to 276	277 to 305	306 to 342	>342	
Person‐years	469 774	462 968	471 581	472 193	471 093	
Total CHD						
Cases	698	736	731	752	697	
Age‐adjusted model	1.0 (ref)	0.91 (0.82 to 1.01)	0.82 (0.74 to 0.91)	0.79 (0.71 to 0.88)	0.70 (0.63 to 0.78)	<0.001
Multivariable model 1*	1.0 (ref)	0.98 (0.87 to 1.10)	0.91 (0.79 to 1.04)	0.90 (0.77 to 1.05)	0.83 (0.69 to 0.98)	0.02
Multivariable model 2*	1.0 (ref)	0.99 (0.87 to 1.11)	0.94 (0.82 to 1.08)	0.95 (0.82 to 1.11)	0.88 (0.74 to 1.04)	0.12
Multivariable model 3*	1.0 (ref)	0.99 (0.88 to 1.12)	0.93 (0.81 to 1.06)	0.95 (0.81 to 1.10)	0.88 (0.74 to 1.05)	0.15
Nonfatal CHD						
Cases	474	522	511	514	490	
Age‐adjusted model	1.0 (ref)	0.97 (0.85 to 1.10)	0.87 (0.77 to 0.99)	0.83 (0.73 to 0.94)	0.76 (0.67 to 0.87)	<0.001
Multivariable model 1	1.0 (ref)	1.04 (0.90 to 1.20)	0.98 (0.83 to 1.15)	0.99 (0.82 to 1.18)	0.97 (0.78 to 1.20)	0.66
Multivariable model 2	1.0 (ref)	1.05 (0.91 to 1.21)	1.01 (0.85 to 1.19)	1.04 (0.86 to 1.24)	1.03 (0.83 to 1.27)	0.88
Multivariable model 3	1.0 (ref)	1.05 (0.91 to 1.21)	0.99 (0.84 to 1.17)	1.02 (0.85 to 1.23)	1.02 (0.82 to 1.25)	0.97
Fatal CHD						
Cases	224	214	220	238	207	
Age‐adjusted model	1.0 (ref)	0.78 (0.65 to 0.94)	0.72 (0.60 to 0.87)	0.71 (0.59 to 0.86)	0.58 (0.48 to 0.70)	<0.001
Multivariable model 1	1.0 (ref)	0.85 (0.69 to 1.06)	0.77 (0.60 to 0.99)	0.74 (0.56 to 0.97)	0.58 (0.42 to 0.79)	<0.001
Multivariable model 2	1.0 (ref)	0.86 (0.69 to 1.07)	0.81 (0.63 to 1.03)	0.79 (0.60 to 1.04)	0.61 (0.45 to 0.84)	0.003
Multivariable model 3	1.0 (ref)	0.85 (0.69 to 1.06)	0.80 (0.62 to 1.02)	0.79 (0.60 to 1.04)	0.64 (0.46 to 0.87)	0.006

CI indicates confidence interval; CHD, coronary heart disease; RR, relative risk; BMI, body mass index; MI, myocardial infarction.

* Test for linear trend estimated by assigning the median value of plasma magnesium in each quartile and modeling this as a continuous variable in regression models.

* RR adjusted for age, calendar year, total calories, smoking, BMI, parental history of MI, alcohol intake, physical activity, menopausal therapy, multivitamin use, and intake of omega‐3 fats, polyunsaturated:saturated fat, trans fat, dietary cholesterol, calcium, cereal fiber, potassium, and vitamin D.

* RR adjusted for covariates in model 1 plus history of hypertension, hypercholesterolemia, and diabetes at baseline.

* RR adjusted for covariates in model 1 plus the development of possible intermediate diseases (hypertension, hypercholesterolemia, and diabetes) during follow‐up.

Magnesium intake was not associated with risk of nonfatal CHD but was significantly inversely associated with risk of fatal CHD (Table 2). The multivariable RR of fatal CHD was 0.61 (95% CI, 0.45 to 0.84) comparing the highest quintile with the lowest quintile of magnesium intake (model 2). This inverse association remained significant after excluding sudden and/or arrhythmic cardiac deaths (n=187; multivariable RR for top quintile, 0.68; 95% CI, 0.48 to 0.96) and when we excluded fatal events based on death certificates alone (n=288; multivariable RR, 0.61; 95% CI, 0.42 to 0.87). When we explored the association between magnesium from food sources only and risk of fatal CHD, results were similar (RR, 0.65; 95% CI, 0.47 to 0.89). We detected no deviation from linearity in the relation between dietary magnesium and risk of total, fatal, and nonfatal CHD. The RR of fatal CHD per a 100 mg/day increment of dietary magnesium was 0.70 (95% CI, 0.56 to 0.87) adjusting for potential confounders (Table 3).

**Table 3.** Mediation Proportion for the Effect of Magnesium Intake on CHD Risk Explained by Hypertension, Diabetes, and High Cholesterol

	RR (95% CI) per 100 mg	Mediation Proportion (95% CI)
Total CHD		
Base model*	0.83 (0.74 to 0.94)	
Base model+hypertension	0.88 (0.78 to 0.99)	29 (11 to 47)
Base model+diabetes	0.85 (0.75 to 0.95)	8 (2 to 14)
Base model+high cholesterol	0.83 (0.74 to 0.93)	N/A*
Fatal CHD		
Base model*	0.70 (0.56 to 0.87)	
Base model+hypertension	0.76 (0.62 to 0.94)	23 (10 to 36)
Base model+diabetes	0.71 (0.57 to 0.87)	3 (−0.9 to 7)
Base model+high cholesterol	0.70 (0.56 to 0.86)	N/A*

CHD indicates coronary heart disease; RR, relative risk; CI, confidence interval; BMI, body mass index; MI, myocardial infarction.

* RR adjusted for age, total calories, smoking, BMI, parental history of MI, alcohol intake, physical activity, menopausal therapy, multivitamin use, and intake of omega‐3 fats, polyunsaturated:saturated fat, trans fat, dietary cholesterol, calcium, cereal fiber, potassium and vitamin D.

* Mediation proportion not calculated because the addition of intermediate variables to the base model did not attenuate the RR.

Next, we explored potential mechanistic pathways through which magnesium intake may lower CHD risk. After adjusting for diabetes, hypertension, and hypercholesterolemia throughout follow‐up, magnesium intake remained significantly associated with risk of fatal CHD (Table 2, model 3). In mediation analyses, the association between magnesium and hypertension accounted for 29% (95% CI, 11% to 47%) and 23% (95% CI, 10% to 36%) of the association between magnesium intake and total and fatal CHD, respectively (Table 3). Diabetes explained 8% (95% CI, 2% to 14%) of the association between magnesium intake on risk of total CHD and was not a mediator for fatal CHD. Self‐reported hypercholesterolemia was not a mediator. Magnesium intake was not significantly associated with risk of nonfatal CHD; thus, we did not estimate the mediation proportion for this outcome.

### Plasma Magnesium Analysis

The characteristics of the population by quartiles of plasma magnesium are presented in Table 4**.** Women with higher plasma magnesium were less likely to have diabetes and use hormone therapy and had higher LDL cholesterol, lower hsCRP, and lower eGFR. Plasma magnesium was not significantly correlated with dietary magnesium (*r*=0.02, *P*=0.50).

**Table 4.** Characteristics in 1990 by Quartile (Q) of Plasma Magnesium in the Total Population

	Plasma Magnesium			
	Q1	Q2	Q3	Q4
Range of magnesium, mg/dL	≤2.0	2.1 to 2.1	2.2 to 2.3	≥2.4
n	164	125	405	222
Age, y	58 (7)*	59 (6)	59 (7)	60 (6)
Current smoker, %	24	23	27	23
History of comorbidities, %				
Hypertension	42	47	34	33
Diabetes	16	10	9	3
Parental history of MI, %	17	13	18	17
BMI, kg/m^2^	26.9 (5.4)	26.4 (5.5)	25.7 (4.6)	25.2 (4.3)
Physical activity, h/week	3.4 (3.3)	3.6 (4.0)	3.6 (4.1)	4.1 (4.0)
eGFR, mL/min per 1.73 m^2^*	88 (20)	84 (21)	84 (18)	82 (18)
Cardiovascular biomarkers				
LDL‐cholesterol, mg/dL	129 (38)	137 (40)	141 (36)	147 (40)
HDL‐cholesterol, mg/dL	55 (18)	57 (16)	56 (16)	57 (16)
C‐reactive protein, mg/dL	0.49 (0.62)	0.39 (0.54)	0.41 (0.76)	0.28 (0.37)
HbA1C, %	5.8 (1.2)	5.7 (1.1)	5.7 (1.0)	5.5 (0.7)
Adiponectin, mg/L	8264 (4117)	8595 (4535)	8631 (3577)	9217 (4088)
Nutrients*				
Magnesium, mg/day	304 (77)	304 (61)	306 (72)	310 (80)
Potassium, mg/day	2904 (536)	2912 (451)	2889 (475)	2924 (518)
Calcium, mg/day	1033 (484)	1033 (514)	1072 (531)	1050 (574)
Alcohol, g/day	5.3 (8.8)	4.0 (6.9)	5.5 (9.7)	5.2 (9.3)
Current use of thiazide diuretics, %	25	23	14	16
Current use of postmenopausal hormones, %	49	43	32	35

MI indicates myocardial infarction; BMI, body mass index; eGFR, estimated glomerular filtration rate; LDL, low‐density lipoprotein; HDL, high‐density lipoprotein; HbA1c, hemoglobin A1c; SD, standard deviation.

* Mean (SD).

* Estimated by the Modification of Diet in Renal Disease Study Group equation.

* All nutrients except alcohol were energy adjusted.

We did not observe a linear inverse association between plasma magnesium and risk of CHD in models adjusting for potential confounders (*P*–test for linear trend=0.22; Table 5, model 2). However, we found a significantly lower risk of CHD in the second quartile of plasma magnesium, with minimal change with higher concentration (RR for magnesium ≥2.1 versus <2.1 mg/dL, 0.65; 95% CI, 0.44 to 0.96). Results were not appreciably altered after further adjustment for magnesium intake and baseline disease status (Table 5), and the L‐shaped association was confirmed in spline analysis (Figure). After adjustment for potential intermediary cardiovascular biomarkers, the association was attenuated and no longer significant (RR for magnesium ≥2.1 versus <2.1 mg/dL, 0.67; 95% CI, 0.44 to 1.04; Table 5). With only 58 cases of fatal CHD, our power to draw any conclusions about the relation between plasma magnesium and fatal CHD separately was limited.

**Table 5.** Relative Risk (95% CI) of CHD by Quartile (Q) of Plasma Magnesium

	Plasma Magnesium					
	Q1	Q2	Q3	Q4	*P*–Linear Trend*	RR Comparing ≥2.1 With <2.1 mg/dL
Range of magnesium, mg/dL	≤2.0	2.1 to 2.1	2.2 to 2.3	≥2.4		
Cases	98	55	204	101		
Controls	66	70	201	121		
Model 1*	1.0 (ref)	0.51 (0.31 to 0.82)	0.65 (0.44 to 0.96)	0.54 (0.35 to 0.82)	0.04	0.59 (0.41 to 0.85)
Model 2*	1.0 (ref)	0.51 (0.30 to 0.85)	0.73 (0.48 to 1.11)	0.61 (0.39 to 0.97)	0.22	0.65 (0.44 to 0.96)
Model 2+magnesium intake	1.0 (ref)	0.50 (0.30 to 0.84)	0.74 (0.49 to 1.12)	0.61 (0.39 to 0.97)	0.23	0.65 (0.44 to 0.96)
Model 2+diabetes, hypertension, and high cholesterol at baseline	1.0 (ref)	0.47 (0.27 to 0.81)	0.76 (0.48 to 1.20)	0.60 (0.37 to 0.98)	0.30	0.65 (0.42 to 0.99)
Model 2+HDL‐C, LDL‐C, hsCRP, adiponectin, and HbA1c	1.0 (ref)	0.50 (0.28 to 0.88)	0.78 (0.49 to 1.25)	0.63 (0.38 to 1.05)	0.37	0.67 (0.44 to 1.04)

CI indicates confidence interval; CHD, coronary heart disease; RR, relative risk; HDL‐C, high‐density lipoprotein cholesterol; LDL‐C, low density lipoprotein cholesterol; hsCRP, high‐sensitivity C‐reactive protein; HbA1c, hemoglobin A1c; BMI, body mass index; MI, myocardial infarction; eGFR, estimated glomerular filtration rate.

* Test for linear trend calculated by assigning the median value of magnesium in each quintile and modeling this as a continuous variable in regression models.

* Model 1: RR estimated from logistic regression models, conditioned on matching factors (age, smoking status, month of blood draw, and fasting status).

* Model 2: RR further adjusted for BMI, exercise, alcohol intake, family history of MI, eGFR, menopausal therapy, multivitamin use, and intake of omega‐3 fats, polyunsaturated:saturated fat, trans fat, dietary cholesterol, cereal fiber, calcium, potassium, and vitamin D.

**Figure 1.**
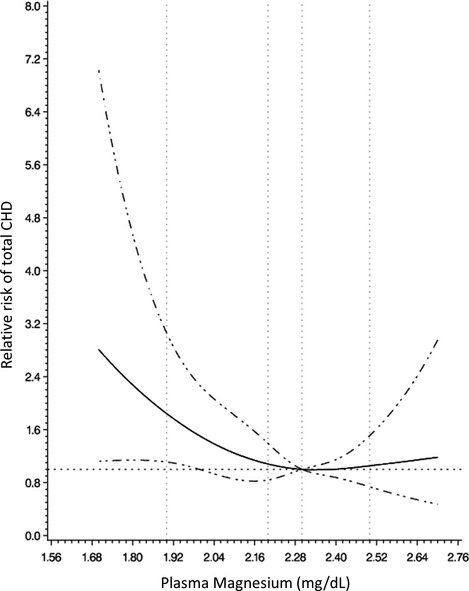
Multivariate relative risk of total CHD as a function of plasma magnesium. Data were fitted by a restricted cubic spline Cox proportional hazards model. The 95% confidence intervals are indicated by dashed lines. Models conditioned on matching factors (age, smoking status, month of blood draw, and fasting status) and adjusted for BMI, exercise, alcohol intake, family history of MI, eGFR, menopausal therapy, multivitamin use, intake of omega‐3 fats, polyunsaturated:saturated fat, trans fat, dietary cholesterol, cereal fiber, calcium, potassium, vitamin D, magnesium, baseline hypertension, baseline hypercholesterolemia, baseline diabetes, and concentration of HDL‐C, LDL‐C, hsCRP, adiponectin, and HbA1c. CHD indicates coronary heart disease; BMI, body mass index; MI, myocardial infarction; eGFR, estimated glomerular filtration rate; HDL‐C, high‐density lipoprotein cholesterol; LDL‐C, low‐density lipoprotein cholesterol; hsCRP, high‐sensitivity C‐reactive protein; HbA1c, hemoglobin A1c.

## Discussion

In this prospective cohort of women, higher magnesium intake was associated with lower risk of fatal CHD, independent of known CHD risk factors. Women with the highest compared with the lowest intake of magnesium had a 39% lower risk of fatal CHD, and this association was mediated partially by hypertension. Dietary magnesium was not associated with nonfatal CHD after controlling for cardiovascular risk factors. Plasma magnesium was associated with risk of CHD in an L‐shaped fashion. Higher concentration of plasma magnesium was associated with lower risk of CHD, although this association was not independent of CVD biomarkers.

Prior studies of dietary magnesium and risk of CHD have yielded conflicting results. Two prospective studies found an inverse association between dietary magnesium and risk of total CHD among men^8,22^; however, in other studies, the association between dietary magnesium and CHD risk has been null.^7,23^ In a meta‐analysis of 5 studies, magnesium intake was not associated with total CHD (RR comparing high with low magnesium intake, 0.86; 95% CI, 0.67 to 1.10).^11^ Consistent with previous studies, we did not find a significant association between dietary magnesium and total CHD risk. In this study, higher dietary magnesium was only associated with lower fatal CHD, and discrepant results in prior studies on total CHD could be due in part to varying proportions of fatal versus nonfatal events in prior studies. The meta‐analysis did not report on the differential relation with fatal and nonfatal events.

For plasma magnesium, we observed an L‐shaped association with risk of CHD, in which individuals with plasma magnesium levels above the second quartile were associated with lower risk of CHD. This nonlinear association of circulating magnesium is consistent with previous relationships for arrhythmic end points, including SCD and atrial fibrillation.^2,24–26^ Low serum magnesium has also been more strongly related to fatal compared with nonfatal CHD^9^; however, we were unable to explore the association between plasma magnesium and fatal CHD separately. In a recent study, serum magnesium was not predictive of total CVD, defined as angina pectoris, CHD, stroke, transient ischemic attack, heart failure, and intermittent claudication.^10^ Any true association with fatal CHD may have been missed with this heterogeneous end point.

Magnesium deficiency has been associated with accelerated atherosclerotic progression,^27–28^ which may lead to more aggressive CHD that is more likely to be fatal. Magnesium has also been associated with beneficial effects on blood pressure,^4^ glucose metabolism,^29^ and lipids. Thus, a novel aspect of this study was the exploration of mediators that might underlie the association between magnesium and risk of CHD. We found that hypertension, but not diabetes or hypercholesterolemia, statistically accounted for part of the association between dietary magnesium and fatal CHD, explaining ~23% of the association. However, even after adjusting for these potential mediators, dietary magnesium remained significantly associated with risk of fatal CHD. The association between plasma magnesium and CHD was attenuated and not significant after adjustment for potential intermediate cardiovascular biomarkers; however, this analysis included mostly nonfatal events. These results suggest that magnesium may prevent fatal CHD through mechanisms beyond known traditional pathways. Magnesium has been also been associated with beneficial effects on inflammation,^30^ endothelial dysfunction,^30^ thrombosis,^31^ and vascular smooth muscle cell calcification,^32^ and alterations in these individual risk factors might influence the propensity for fatal events.

In addition, magnesium has antiarrhythmic effects,^3,33^ and chronic magnesium deficiency may be proarrhythmic.^34–35^ In epidemiological studies, low serum magnesium has been associated with higher risk of atrial fibrillation^24–25^ and SCD,^2,26^ further supporting a direct link between hypomagnesemia and cardiac arrhythmias. In this study, the strong inverse association for fatal CHD persisted even after the exclusion of known SCD events. Because the circumstances surrounding the death are not always known, a proportion of the fatal CHD events that did not fulfill the strict definition for SCD^36^ may have been arrhythmic in origin. However, these unidentified events are unlikely to account for the entire association with fatal CHD. Therefore, antiarrhythmic actions of magnesium are unlikely to account entirely for the association observed. Nevertheless, the lack of ECG data limits our ability to explore the mediation effects of electrocardiographic intervals.

Clinical trial data regarding magnesium supplementation in the secondary prevention of CHD are sparse. In several large‐scale randomized trials, intravenous magnesium in the setting of an acute MI did not reduce mortality rates and low‐dose oral magnesium among 468 acute MI survivors did not lower risk of cardiac events over 1 year.^37^ No clinical trials to date have assessed magnesium supplementation for the primary prevention of CHD. It is plausible that only individuals with low baseline intake may benefit from supplementation.^38^ Given that the current average magnesium intake in the United States is 261 mg in women and 347 mg in men, which is below the established RDA (420 and 320 mg/day in men and women >30 years old, respectively),^39^ magnesium supplementation or higher magnesium diets might be beneficial for the primordial prevention of CHD in the US population. Although plasma magnesium is a poor biomarker of magnesium intake, as evident by the lack of correlation with dietary magnesium, low‐magnesium diets may lead to low serum magnesium levels.^40^ In addition, magnesium supplementation can significantly increase magnesium stores,^41^ particularly among individuals with hypomagnesaemia at baseline.^42^ Ultimately, only a long‐term clinical trial of magnesium supplementation in persons with low dietary intakes with CHD as the end point can determine the true effect of magnesium supplementation on CHD prevention.

Our study has several important limitations. First, given the relatively small number of events in the nested case–control analysis, we had limited power to explore the association between serum magnesium and fatal CHD. Second, multivitamins and foods that are major sources of magnesium, such as whole grains, fruits, vegetables, and nuts, contain many other nutrients that may lower CHD risk. Therefore, although we controlled for many variables in our dietary models, residual confounding by nutrients such as calcium or potassium may explain part of the association between magnesium and risk of CHD. Third, we used self‐reported measures of potential mediators, as we lacked direct measures of blood pressure, cholesterol, and glucose in most women in the cohort analysis and therefore may not have captured the full mediation effects of these traditional risk factors on the association between dietary magnesium and CHD risk. Further, we lacked data on other biomarkers of inflammation, endothelial dysfunction, and thrombosis in the majority of women and ECG data in all women to assess other mediation pathways. In the analysis of plasma magnesium, we adjusted for prevalent hypertension, high cholesterol, and diabetes and directly measured lipids and inflammatory biomarkers. Because these factors were assessed at the same time as plasma magnesium, we cannot determine whether low plasma magnesium was a cause or consequence of elevated biomarkers. Therefore, these models may overcontrol for biologic intermediates.

In conclusion, higher magnesium intake was not associated with overall CHD risk after controlling for CHD risk factors, but was associated with a lower risk of fatal CHD. This latter association was only partially explained by traditional CHD risk factors. In addition, plasma magnesium levels above the second quartile were associated with lower risk of CHD, although not independent of other cardiovascular biomarkers. Future studies exploring the differential association between plasma magnesium and fatal and nonfatal CHD events are needed. The stronger association of magnesium intake and risk of fatal CHD and the lack of association between magnesium and non‐fatal CHD in the current study, in combination with our prior finding on SCD, further supports the hypothesis that antiarrhythmic and other actions of magnesium may reduce the risk of fatal CHD.
